# Toward Spectroscopic Accuracy for the Structures of Large Molecules at DFT Cost: Refinement and Extension of the Nano-LEGO Approach

**DOI:** 10.1021/acs.jpca.3c01617

**Published:** 2023-06-07

**Authors:** Vincenzo Barone, Giorgia Ceselin, Federico Lazzari, Nicola Tasinato

**Affiliations:** Scuola Normale Superiore, Piazza dei Cavalieri 7, I-56126, Pisa, Italy

## Abstract

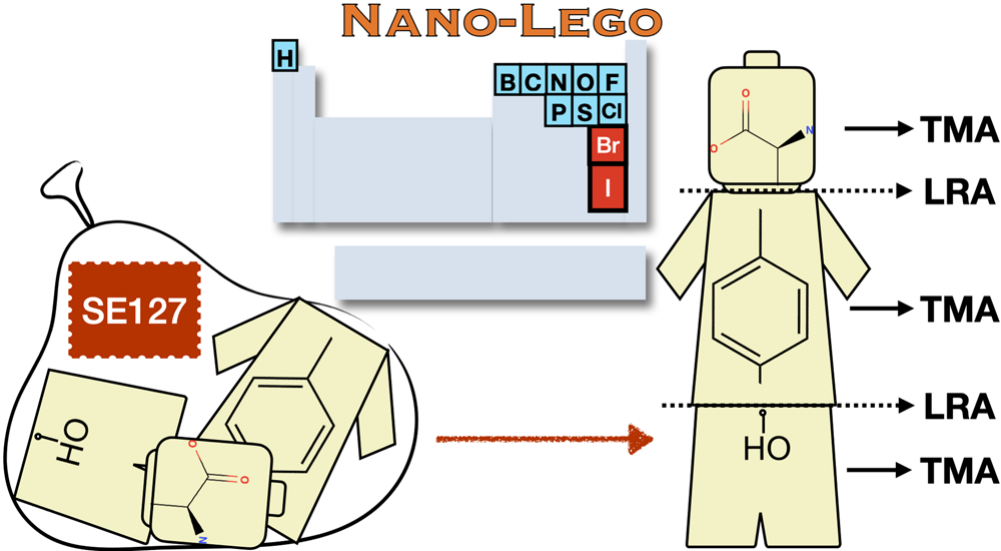

The SE100 database collecting accurate equilibrium geometries of medium size molecules obtained by the semiexperimental (SE) approach has been extended to species containing Br and I atoms. This has allowed the determination of accurate linear regressions between DFT and SE values for all the main bonds and angles involving H, B, C, N, O, F, P, S, Cl, Br, and I atoms. An improved Nano-LEGO tool has been developed, which is based on suitable hybrid and double hybrid functionals and combines in a fully coherent way the templating molecule and linear regression approaches. A number of case studies show that the new Nano LEGO tool provides geometrical parameters on par with state-of-the-art composite wave function methods, but can be routinely applied to medium- to large-size molecules. The accuracy reached for structural parameters is mirrored on rotational constants that can be predicted with an average error within 0.2%.

## Introduction

Several fields of molecular sciences make extensive use of structure–property relationships, which are the result of a subtle balance among a number of intra- and intermolecular interactions. Gas-phase studies are, therefore, mandatory in order to characterize intrinsic stereoelectronic effects, which are then tuned by interactions with the environment. In the case of large molecules, the complexity of molecular topology and the flexibility of both the backbone and side chains add the additional difficulty of disentangling local and nonlocal intramolecular interactions. This has stimulated several experimental and computational studies of flexible molecules in the gas-phase, which are providing a detailed knowledge of their structures and conformational landscapes. In particular, the coupling of supersonic-jet expansion^1^ and laser ablation^2^ has allowed the recording of gas-phase microwave (MW) spectra for thermolabile species, like most bricks of life.^3^ However, the fast relaxation of some structures to more stable counterparts in the presence of low energy barriers and the photodissociation of some products can bias any direct thermochemical interpretation of the results provided by this technique.^4,5^

Quantum chemical (QC) computations can be profitably used to solve this kind of problems, but the increasing dimensions of the molecules amenable to high-resolution spectroscopic studies and the need of characterizing several different structures (e.g., conformers or tautomers)^6,7^ exacerbate the never ending fight between accuracy and feasibility. Furthermore, conventional local optimization techniques are very powerful for semirigid systems, but cannot be applied to the exploration of flat potential energy surfaces (PESs).^8,9^ We have recently developed an integrated computational approach combining different QC methods driven by machine learning (ML) tools for an effective exploration of conformational PESs and the successive refinement of the most significant stationary points.^8,10−12^ Once the final panel of low-energy minima has been defined, their relative stability and spectroscopic parameters need to be computed at high accuracy to allow an unbiased reproduction and interpretation of experimental results. Thanks to ongoing developments, state-of-the-art methods rooted in the density functional theory (DFT) are offering a very effective compromise between reliability and scaling with the dimensions of the investigated system. In particular, the accuracy of the structural parameters delivered by some hybrid and (especially) double-hybrid functionals in conjunction with (partially) augmented medium-size basis sets is largely sufficient for most applications.^13,14^ However, rotational constants (the leading terms of MW spectra) require more accurate geometrical parameters, which can be obtained only by means of state-of-the-art composite wave function methods.^15−17^ Several studies have shown that very accurate molecular structures can be obtained leaving unchanged the valence and dihedral angles provided by DFT methods and that the systematic nature of the errors permits significant improvement of the bond length values by a linear regression approach (LRA).^18−20^ In this model, the bond length (*r*_*M*_) between two atoms of type X and Y in the studied molecule (M) is obtained from the value computed by a DFT method *r*_*M*_^*DFT*^ by means of scaling factors (*a*_*XY*_) and offset parameters (*b*_*XY*_) depending on the nature of the involved atoms:
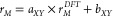
1

In order to determine the *a*_*XY*_ and *b*_*XY*_ parameters, a large database containing 100 semi experimental (SE) equilibrium structures (SE100) has been built and made available to the scientific community.^18^ Instead of employing an overall linear regression, one can resort, when possible, to a templating molecule (TM) sharing structural similarities with the species under study and whose SE equilibrium structure is already available. Within this approach, the geometry of each fragment can be obtained as
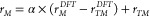
2with eq 1 being used for interfragment parameters. The original templating molecule approach (TMA)^21,22^ employed α = 1 for all the intrafragment bond lengths, whereas we now suggest using, whenever possible, α = a_*XY*_ in order to make LRA and TMA fully consistent. The choice of the most suitable templating molecule can be performed on the basis of chemical intuition, but work is in progress toward a full automation of fragment recognition by means of machine learning tools employing suitable descriptors.^23^

Based on these premises, the main goals of the present study are (i) to further extend the SE100 database^18^ mostly (but not exclusively) with molecules containing Br and I atoms; (ii) to integrate in a fully coherent way the LRA and TMA models, and (iii) to show by means of a number of case studies that the new Nano-LEGO tool can be routinely used for obtaining geometries (hence rotational constants) with an accuracy comparable to that delivered by state-of-the-art quantum chemical methods for small semirigid molecules.

## Methods

Equilibrium molecular structures were obtained by using the SE approach first introduced by Pulay and co-workers.^24^ In practice, SE rotational constants (*B*_*SE*_^*i*^, where *i* denotes the inertial axis *a*, *b*, or *c*) can be obtained by removing vibrational contributions (*ΔB*_*vib*_^*i*^) from the corresponding ground state rotational constants (*B*_0_^*i*^) measured experimentally. Vibrational perturbation theory to second-order (VPT2)^25−28^ permits obtaining analytical (resonance-free) expressions for *ΔB*_*vib*_^*i*^ including contributions from harmonic force constants, Coriolis couplings, and semidiagonal cubic force constants. From a quantitative point of view, the *ΔB*_*vib*_^*i*^ terms are usually well below 1% of the corresponding equilibrium rotational constants,^29^ so that they can be safely determined by DFT methods, which deliver typical errors within 10% (i.e., less than 0.1% of typical rotational constants).^18,21,22,30^ Then, SE equilibrium rotational constants of different isotopologues are used in a nonlinear least-squares fit for obtaining SE equilibrium geometrical parameters.

Quantum chemical calculations were carried out by using hybrid and double-hybrid density functionals, which deliver remarkably accurate structural and spectroscopic properties.^7,14,20,31^ In particular, the PW6B95 hybrid density functional^32^ was employed in conjunction with the jul-cc-pVDZ partially augmented double-ζ basis set,^33^ whereas the rev-DSDPBEP86 double-hybrid density functional^34^ was used in conjunction with the jun-cc-pVTZ basis set.^33^ Noted is that the B2PLYP double-hybrid functional^35^ provides predictions of comparable accuracy for geometries, rotational spectroscopic parameters, and vibrational properties^22,36−39^ when used in conjunction with triple-ζ basis sets. Empirical dispersion corrections were added to both PW6B95 and rev-DSDPBEP86 density functionals by means of the Grimmes D3 scheme^40^ with Becke–Johnson damping (D3BJ).^41^ Since tight *d* functions are important for a quantitative representation of the electronic structure of second-row elements,^42^ partially augmented basis sets, namely jul-/jun-cc-pV(*n* + *d*)Z with *n* = D, T, including an additional set of tight *d* functions, were employed for sulfur and chlorine atoms. For the Br and I atoms, the aug-cc-pVDZ-PP and aug-cc-pVTZ-PP basis sets and the corresponding small-core pseudopotentials^43,44^ were used in the calculations carried out with the PW6B95 and rev-DSDPBEP86 functional, respectively. The overall computational models (density functional, basis set and, possibly, effective core potentials) will be denoted in the following as PW6 and rDSD, respectively.

After full geometry optimization at each level of theory, analytical Hessians were computed and employed to obtain by numerical differentiation the semidiagonal cubic force constants needed for the evaluation of vibrational contributions to rotational constants in the framework of VPT2.^25−27^

The Gaussian16 suite of programs^45^ was employed for all the DFT calculations and the MSR software^46^ for SE fittings. For reasons explained in the following, some MP2 computations were performed by utilizing the CFOUR package.^47^

## Results and Discussion

### Equilibrium Geometries of Br-Containing Molecules

**Figure 1:**
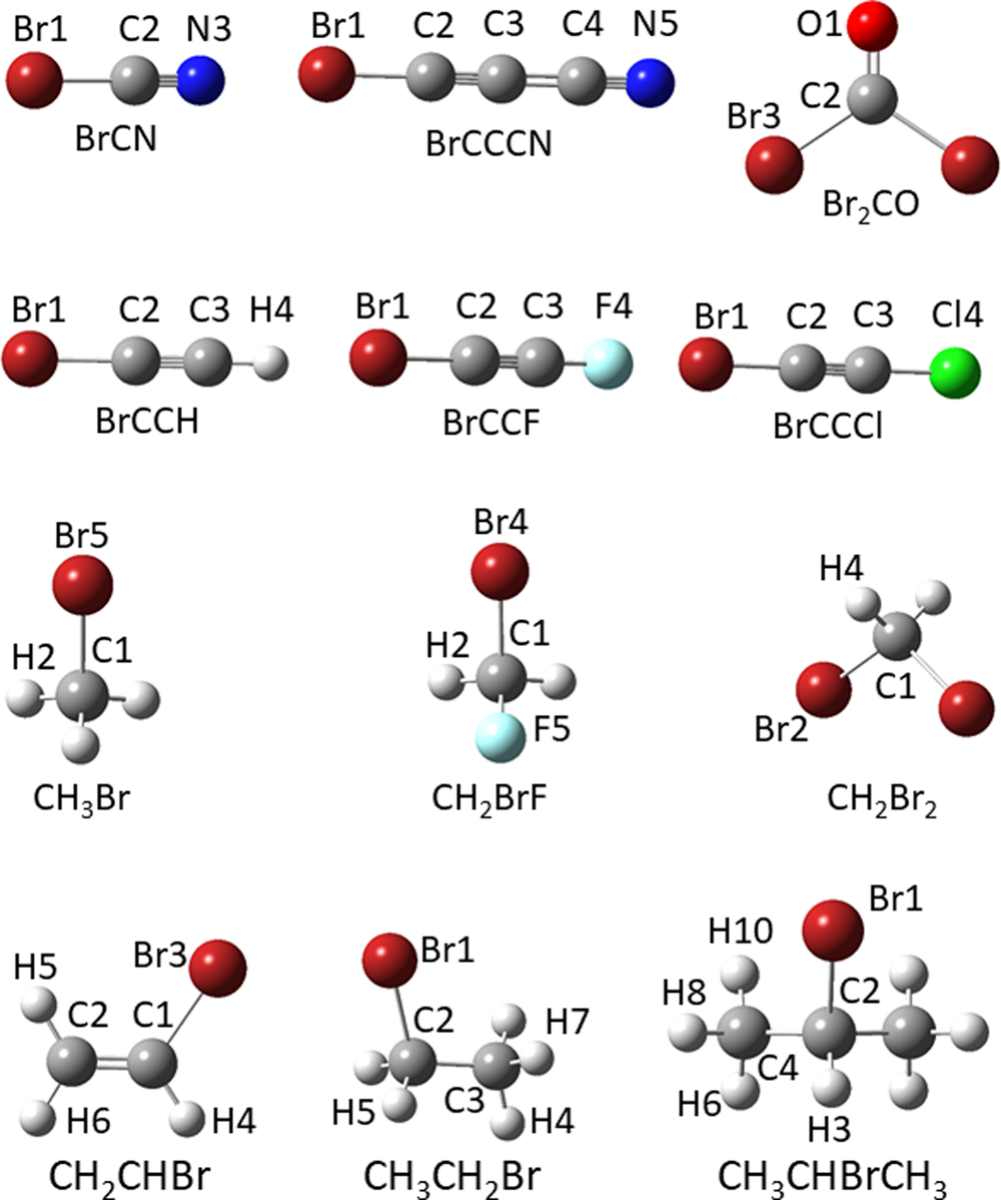
Molecular structures and atom-labeling of the organo-bromine molecules for which SE equilibrium structures are available or have been computed in this work.

The 12 organo-bromine molecules shown in Figure 1 (BrCN, Br_2_CO, BrCCH, BrCCF, BrCCCl, BrCCCN, CH_3_Br, CH_2_Br_2_, CH_2_BrF, CH_2_CHBr, CH_3_CH_2_Br, CBrH(CH_3_)_2_) have been selected for characterizing the C–Br bond.

The SE equilibrium structures of BrCN,^48^ CH_3_Br,^49^ CH_2_BrF,^50^ and CH_2_CHBr^51^ have been taken from the literature, and they can be found in Table S1 of the Supporting Information (SI), whereas the SE equilibrium structures of the remaining eight species have been determined (or redetermined) in the present work.

The SE equilibrium structures of the four linear molecules are reported in Table 1, together with the equilibrium structures predicted at the PW6 and rDSD levels of theory. Since linear molecules including heavy atoms are strongly sensitive to numerical errors, the vibrational corrections for those species have been computed using second-order Møller–Plesset perturbation theory (MP2)^52^ in conjunction with cc-pVTZ and cc-pVTZ-PP basis sets.

**Table 1:** SE, rDSD, and PW6 Equilibrium Geometries of Linear Molecules Containing Br Determined in the Present Work^a^

**Molecule**	**Parameter**	**SE**	**rDSD**	**PW6**
BrC≡CH	*r*(Br1–C2)	1.7897(1)	1.7921	1.7918
	*r*(C2≡C3)	1.2036(1)	1.2080	1.2083
	*r*(C3–H4)	1.0617(1)	1.0637	1.0680
				
BrC≡CF	*r*(Br1–C2)	1.7913(1)	1.7952	1.7951
	*r*(C2≡C3)	1.1980^b^	1.2002	1.2006
	*r*(C3–F4)	1.2763^b^	1.2802	1.2802
				
BrC≡CCl	*r*(Br1–C2)	1.7970(18)	1.7910	1.7909
	*r*(C2–C3)	1.1935(28)	1.2083	1.2086
	*r*(C3–Cl4)	1.6367(12)	1.6375	1.6326
				
BrC≡CC≡N	*r*(Br1–C2)	1.77982	1.7804	1.7780
	*r*(C2≡C3)	1.2073	1.2130	1.2128
	*r*(C3–C4)	1.37312	1.3728	1.3695
	*r*(C4≡N5)	1.15980	1.1665	1.1618

a Bond lengths in Å. Standard deviations in the units of the last significant digits are given in parentheses.

b Fixed at rDSD+Nano-LEGO value.

The SE equilibrium structure of bromoacetylene has been determined by using the rotational constants of 12 isotopic species (H^12^C^12^C^79^Br, H^12^C^13^C^79^Br, H^13^C^12^C^79^Br, D^12^C^12^C^79^Br, D^12^C^13^C^79^Br, H^13^C^12^C^79^Br, and their ^81^Br- isotopologues), which were originally employed to derive the substitution *r*_*s*_ structure.^53^ Inspection of Table 1 shows that all the equilibrium geometrical parameters are well determined, with statistical errors on bond lengths around 0.02 mÅ, that have been rounded to 0.1 mÅ to give a more realistic estimate of the expected error. In addition to the equilibrium structure, the effective *r*_0_ geometry has been retrieved by fitting the structural parameters of the molecule to the ground state rotational constants of the same set of isotopologues, obtaining *r*_0_(C–Br) = 1.7923 Å, *r*_0_(C≡C) = 1.2034 Å, and *r*_0_(C–H) = 1.0552 Å. Comparison of these values with their equilibrium counterparts points out a significant overestimation (≈ 3 mÅ) of the C–Br bond length and an even larger underestimation (≈ 6 mÅ) of the C–H bond length, highlighting the significant contributions of vibrational effects to the effective ground state geometry. The *r*_*s*_ structure determined by Jones at al.^53^ is in good agreement with our *r*_0_ structure, and therefore, it shows similar deviations from the equilibrium molecular configuration. At the same time, the SE equilibrium geometry is in remarkable agreement with the equilibrium structure determined from scaled ground-state moments of inertia.^54^

The SE equilibrium geometry of BrCCF has been determined using the ground state rotational constants of the ^79^Br and ^81^Br isotopologues. Due to the lack of isotopic substitutions on C and F atoms, the C≡C and C–F bond lengths have been fixed at their rDSD+Nano-LEGO values. With these constraints, the structural refinement led to a C–Br bond length of 1.7913 Å, in good agreement with the value computed at the CCSD(T)/TZ2Pf level by Breidung et al.^55^

All the SE equilibrium bond lengths of bromochloroacetylene appear well determined, with the largest uncertainty (2.8 mÅ) affecting the C≡C distance. This is understandable by considering that the SE approach has been exploited on the basis of four isotopic species (^79^BrCC^35^Cl, ^79^BrCC^37^Cl, ^81^BrCC^35^Cl, and ^81^BrCC^37^Cl), whose rotational constants were determined from the analysis of MW spectra of the sample in natural abundance.^56^ Also for this molecule, the ground state geometry has been determined in addition to the equilibrium molecular structure, obtaining the following bond lengths: *r*_0_(C–Br) = 1.7872(26) Å, *r*_0_(C≡C) = 1.2116(40) Å, and *r*_0_(C–Cl) = 1.6278(27) Å. These values coincide, within the quoted uncertainties, with the effective *r*_0_ parameters determined in ref (56) and are quite different (0.01–0.02 Å) from the equilibrium values.

The SE equilibrium geometry of bromo-cyano-acetylene has been obtained by correcting the ground state rotational constants determined experimentally for ten isotopic species (^79^BrCCCN, ^79^Br^13^CCCN, ^79^BrC^13^CCN, ^79^BrCC^13^CN, ^79^BrCCC^15^N, and the corresponding isotopologues containing ^81^Br)^57^ with vibrational contributions evaluated theoretically. The fit of the four bond lengths to the SE rotational constants converged with a standard deviation of 4.3 × 10^–3^ uÅ^2^, and all of them appear well determined, with the largest uncertainty (1.1 mÅ) affecting the C≡C bond length.

The SE equilibrium structures of nonlinear molecules are collected in Tables 2 (Br_2_CO, CH_2_Br_2_, and CH_3_CH_2_Br) and 3 (CHBr(CH_3_)_2_), together with the equilibrium structures predicted at the PW6 and rDSD levels of theory. In these cases, the vibrational corrections have been computed at the rDSD level.

**Table 2:** SE, rDSD, and PW6 Equilibrium Geometries of C1 and C2 Nonlinear Molecules Containing Br Determined in the Present Work^a^

**Molecule**	**Parameter**	**SE**	**rDSD**	**PW6**
Br_2_C=O	*r*(O1=C2)	1.17401^b^	1.1775	1.1731
	*r*(C2–Br3)	1.9171(1)	1.9171	1.9209
	α(Br3C2O1)	123.79(1)	123.87	123.72
				
CH_2_Br_2_	*r*(C1–Br2)	1.92178(14)	1.9277	1.9323
	*r*(C1–H4)	1.08035(54)	1.0831	1.0874
	α(Br2C1Br3)	112.862(13)	112.94	113.44
	α(H4C1H5)	112.412(84)	112.37	112.50
				
CH_3_CH_2_Br	*r*(Br1–C2)	1.9479(1)	1.9550	1.9629
	*r*(C2–C3)	1.5098(1)	1.5136	1.5071
	*r*(C3–H4)	1.0911(1)	1.0940	1.0970
	*r*(C2–H5)	1.0844(1)	1.0873	1.0909
	*r*(C3–H7)	1.0868(1)	1.0904	1.0933
	α(C3C2Br1)	111.01(1)	111.01	111.51
	α(H4C2C3)	109.080(12)	109.39	109.14
	α(H5C2C3)	112.60(1)	112.52	112.61
	α(H7C3C2)	109.080(12)	109.39	109.14
	δ(H5C2C3Br1)	117.85(1)	117.86	117.88
	δ(H7C3C2H4)	119.63(1)	119.66	119.47

a Bond lengths in Å, angles in degrees. Standard deviations in the units of the last significant digits are given in parentheses.

b Fixed at rDSD+Nano-LEGO value.

**Table 3:** SE, rDSD, and PW6 Equilibrium Geometries of (CH_3_)_2_CHBr^a^

**Molecule**	**Parameter**	**SE**	**rDSD**	**PW6**
(CH_3_)_2_CHBr	*r*(Br1–C2)	1.96334(29)	1.9702	1.9845
	*r*(C2–H3)	1.08761(64)	1.0892	1.0924
	*r*(C2–C4)	1.51247(24)	1.5163	1.5106
	*r*(C4–H6)	1.09098(98)	1.0945	1.0973
	*r*(C4–H8)	1.0883(11)	1.0899	1.0923
	*r*(C4–H10)	1.08867(61)	1.0917	1.0943
	α(Br1C2H3)	103.584(41)	103.389	102.756
	α(Br1C2C4)	108.962(16)	108.952	108.953
	α(C2C4H6)	109.07(12)	109.206	109.067
	α(C2C4H8)	111.321(72)	111.441	111.490
	α(C2C4H10)	110.309(30)	110.558	110.856
	δ(C4C2Br1H3)	118.01(24)	117.61	117.95
	δ(C2C4H6Br1)	–177.80(20)	–177.92	–177.40
	δ(H8C4C2H6)	–119.94(23)	–119.15	–119.20
	δ(H10C4C2H6)	119.24(20)	119.71	119.95

a Bond lengths in Å, angles in degrees. Standard deviations in the units of the last significant digits are given in parentheses.

Both the equilibrium and the ground state structure of Br_2_CO have been determined for the first time by considering the ground state rotational constants measured by Carpenter et al. for the ^79^Br_2_CO, ^81^Br_2_CO, and ^79^Br^81^BrCO isotopologues.^58^ Here the difficulty in obtaining reliable structural parameters is related to the lack of isotopic substitution for the C=O moiety that, furthermore, lies along the *b* principal axis of inertia. In order to circumvent this problem, the C=O bond length has been fixed to the value obtained by the rDSD+Nano-LEGO approach and only the C–Br bond length and the BrCO valence angle have been refined. Inspection of Table 2 shows that both structural parameters are determined very well and, furthermore, the equilibrium value of the C–Br bond length shows a good correlation with that computed at both rDSD and PW6 levels of theory (vide infra) with this confirming, *a posteriori*, the reliability of the SE equilibrium geometry. For completeness, we recall that the C–Br bond length and the BrCO valence angle of the *r*_0_ structure are 1.9157 Å and 123.82°.

The ground state rotational constants of four dibromomethane isotopic species (CH^79^_2_Br_2_, CH^81^_2_Br_2_, CD^79^_2_Br_2_, CD^81^_2_Br_2_)^59^ have been used to derive its SE equilibrium geometry with the constraint of *C*_2*v*_ symmetry. The fit converged with a standard deviation of about 0.02 u Å^2^ and errors on the retrieved parameters lower than 0.5 mÅ and 0.08° for bond lengths and valence angles, respectively. Comparison of the SE equilibrium geometry with that estimated by Davis and Gerry^59^ shows a good overall agreement, especially concerning valence angles, while the C–H and C–Br bond lengths are over- and underestimated by about 4 and 2 mÅ, respectively.

Finally, the equilibrium geometries of ethyl-bromide and isopropyl-bromide have been fitted using the rotational constants of 13 and 7 isotopologues, respectively.^60,61^ In both cases, the structural parameters (see Tables 2 and 3) appear well determined: indeed, in the case of ethyl-bromide the statistical uncertainties are lower than 0.1 mÅ for bond lengths (rounded to a more conservative estimate of 0.1 mÅ) and around 0.01° for valence and dihedral angles. In the case of isopropyl-bromide (see Figure 1 for atom labeling), the largest uncertainties for the stiff degrees of freedom concern the C4–H8 bond length (1.1 mÅ) and the C2C4H8 valence angle (0.07 degrees), whereas the error is around 0.2° for all dihedral angles. Comparison of the SE equilibrium geometry of CH_3_CH_2_Br with the corresponding substitution structure (*r*_*s*_) reported in ref (60) shows that the latter is affected by larger uncertainties (about one-order of magnitude) in the bond lengths and furthermore significant deviations from the actual equilibrium geometry can be appreciated (up to 0.01 Å for the C–C bond length), with this highlighting once again the need to take vibrational contributions into proper account.

### Equilibrium Geometries of I-Containing Molecules

**Figure 2:**
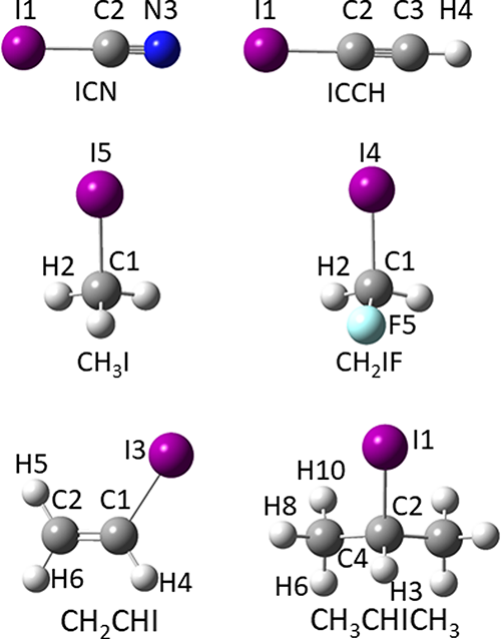
Molecular structures and atom-labeling of the organo-iodine molecules included in the SE127 database.

The SE equilibrium geometries of the six organo-iodine molecules shown in Figure 2 (ICN, CH_3_I, CH_2_FI, HCCI, CH_2_CHI, and (CH_3_)_2_CHI) have been used to characterize the C–I bond. The corresponding SE equilibrium geometries are collected in Table S2 of the SI together with those computed at the rDSD and PW6 levels of theory.

The SE equilibrium structures of ICN,^48^ CH_3_I,^49^ CH_2_FI,^62^ HCCI,^49^ and CH_2_CHI^63^ have been taken from the literature, whereas that of (CH_3_)_2_CHI has been determined in the present work by correcting the ground-state rotational constants determined for 12 isotopic species by vibrational corrections computed at the rDSD level. The resulting structure is detailed in Table 4.

**Table 4:** SE, rDSD, and PW6 Equilibrium Geometries of Isopropyl-iodine^a^

**Parameter**	**SE**	**rDSD**	**PW6**
*r*(I1–C2)	2.1670(5)	2.1747	2.1976
*r*(C2–H3)	1.0909(12)	1.0891	1.0922
*r*(C2–C4)	1.5136(3)	1.5179	1.5111
*r*(C4–H6)	1.1005(7)	1.0953	1.0983
*r*(C4–H8)	1.0943(10)	1.0900	1.0924
*r*(C4–H10)	1.0925(10)	1.0918	1.0944
α(I1C2H3)	102.582(62)	102.671	101.943
α(I1C2C4)	109.451(23)	109.367	109.219
α(C2C4H6)	108.536(96)	109.023	108.881
α(C2C4H8)	111.04(11)	111.766	111.784
α(C2C4H10)	110.395(37)	110.807	111.116
δ(I1C2C4H3)	117.655(27)	117.790	117.456
δ(C2C4H6I1)	–177.11(14)	–177.887	–177.437
δ(C2C4H8H6)	–119.40(12)	–119.022	–118.955
δ(C2C4H10H6)	119.32(14)	119.891	119.643

a Bond lengths in Å, angles in degrees. Standard deviations on the last significant digits are given in parentheses.

All the parameters appear well determined and the agreement between SE and rDSD angles is remarkable.

### The SE127 Structural Database

The SE equilibrium structures of the 12 organobromine and 6 organoiodine molecules discussed in the two preceding sections have been added to our structural database together with those of 9 other molecules (1,3,4-oxadiazole,^64^ H_2_O_2_,^65^ benzonitrile and phenylacetilene,^66^ H_2_C=O–O,^67^ glycolic acid,^7^ 1-chloro-2-fluoroethene,^68^ 1-chloro-2,2-difluoroethene,^69^ aminoacetonitrile^70^). Furthermore, the SE equilibrium structures of 3 molecules already present in the SE100 database (pyrimidine, pyridazine, and thiophene) have been revised employing improved experimental data.^71−73^ The updated database, now containing 127 different molecules, will be referred to as SE127.

**Figure 3:**
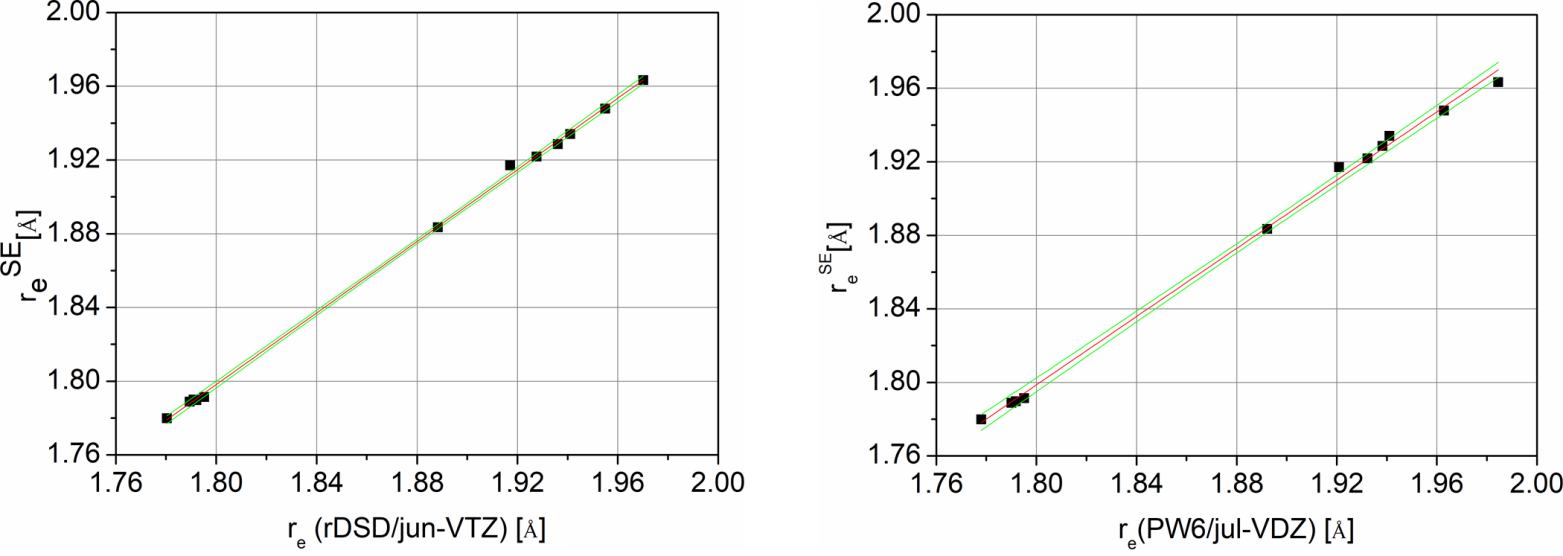
LRA fits for C–Br bond lengths at rDSD (left) and PW6 (right) levels of theory; 95% confidence intervals are also shown (green lines).

**Figure 4:**
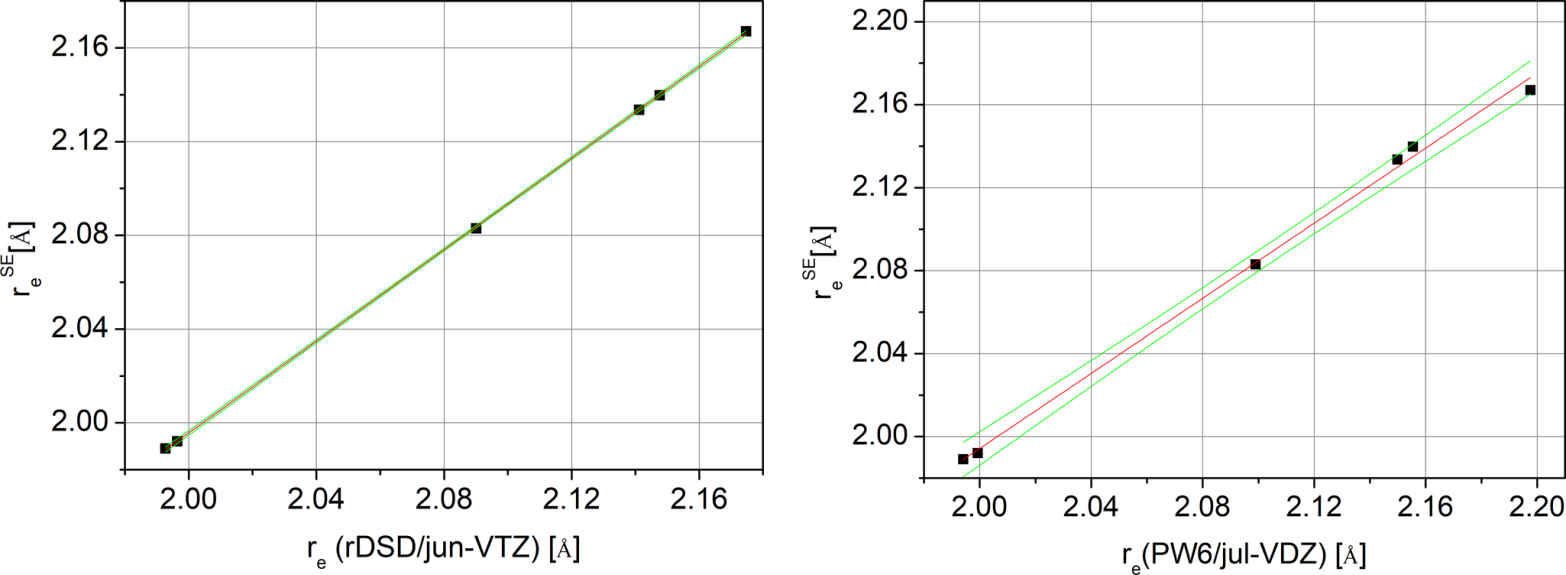
LRA fits for C–I bond lengths at rDSD (left) and PW6 (right) levels of theory; 95% confidence intervals are also shown (green lines).

The rDSD and PW6 predictions against the corresponding SE values, shown in Figure 3 for C–Br bond lengths and in Figure 4 for C–I bond lengths, confirm the expected linear relationships for both functionals. Therefore, the SE127 database has allowed LRA parameters to be obtained for the CX (X = C, N, O, F, S, Cl, Br, I), NX (X = N, O, S), OX (X = O, S), SS, and YH (Y = C, N, O, S) bond lengths collected in Table 5, which can be used within eqs 1 and 2 to improve the accuracy of rDSD and PW6 structural parameters. At the rDSD level all the *a*_*XY*_ parameters are very close to 1.0 and most of the *b*_*XY*_ parameters vanish. Furthermore, the mean absolute errors (MAE) for all bond lengths after the LRA correction are below 1.5 mÅ, whereas for the bare functional the MAE can reach 7.5 mÅ (for the CCl bond). The general trend is similar for the PW6 functional, although the range spanned by the *a*_*XY*_ values and the number of non negligible *b*_*XY*_ parameters become much larger. In general, valence angles can be safely left uncorrected at the rDSD level, whereas this is not the case at the PW6 level.^18^ In this connection we point out that the *a*_*XY*_ employed in eq 1, eq 2, and Table 5 correspond to 1 – *a*_*XY*_ when the notation of ref (18) is used. A last remark concerns the equilibrium SE OO bond length of the simplest Criegee intermediate (H_2_C=O–O),^67^ which falls outside its LRA value at both rDSD and PW6 levels due to the strong zwitterionic character of this molecule. However, this fragment can be confidently used to obtain accurate structures of larger Criegee intermediates when employed as templating molecule in the new Nano-LEGO approach.

**Table 5:** LRA Parameters for rDSD and PW6 Levels of Theory

X–Y	*a*_*XY*_(rDSD)	*b*_*XY*_(rDSD)^a^	*a*_*XY*_(PW6)	*b*_*XY*_(PW6)^a^
C–C	0.99816	0.00000	1.00014	0.00000
C–N	0.99766	0.00000	1.01705	–0.02079
C–O	0.99703	0.00000	1.01708	–0.02120
C–F	0.99693	0.00000	0.99402	0.00000
C–S	0.98778	0.01672	0.98704	0.02188
C–Cl	0.99570	0.00000	0.99860	0.00000
C–Br	0.97099	0.05037	0.92840	0.12753
C–I	0.97680	0.04213	0.97984	0.03562
N–N	0.99880	0.00000	1.00295	0.00000
N–O	0.99950	0.00000	1.00850	0.00000
N–S	0.99553	0.00000	0.99555	0.00000
O–O	1.00158	0.00000	1.01686	0.00000
O–S	0.99349	0.00000	0.98790	0.00000
S–S	0.99580	0.00000	0.99613	0.00000
C–H	0.99761	0.00000	0.99414	0.00000
N–H	0.99784	0.00000	0.99669	0.00000
O–H	0.99640	0.00000	1.17529	–0.17005
S–H	0.99830	0.00000	0.99306	0.00000

a In Å.

### Consistent LRA and TMA Procedures

In order to illustrate the improvement in the equilibrium structures obtained through the Nano-LEGO correction over the bare DF predictions, and to demonstrate how this is mirrored in the accuracy of the corresponding rotational constants, the procedure will be applied to five test cases, which have been selected simply on the basis of the availability of experimental ground state rotational constants. Indeed, in this section, the reliability of the Nano-LEGO correction, as well as of the LRA parametrizations derived for both C–Br and C–I bonds, will be assessed *a posteriori* by inspecting the improved accuracy delivered for the rotational constants. For the purpose, we have adopted the following procedure: first, the equilibrium geometries obtained from the bare functionals have been corrected through the Nano-LEGO approach, by using either the LRA or a combination of the LRA and the new version of the TMA. Then, the equilibrium rotational constants corresponding to the Nano-LEGO corrected equilibrium structures have been derived and augmented by vibrational contributions computed at the PW6 level.

**Figure 5:**
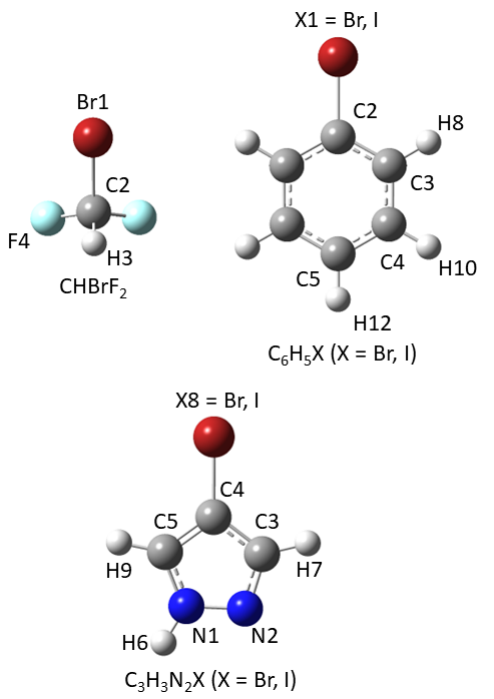
Molecular structures and atom-labeling of bromodifluoromethane (CHBrF_2_), bromobenzene (C_6_H_5_Br), iodobenzene (C_6_H_5_I), 4-bromo-pyrazole (C_3_H_3_N_2_Br), and 4-iodo-pyrazole (C_3_H_3_N_2_I).

The first molecule of the test-set is bromodifluoromethane (CHBrF_2_) (see Figure 5), whose rDSD and PW6 equilibrium geometries are collected in Tabble S3 of the SI together with those obtained after the Nano-LEGO correction. Equilibrium and ground state rotational constants calculated as described above are compared in Table 6 with the values determined experimentally from the analysis of rotational spectra.^74^ The improved accuracy of the results stemming from the Nano-LEGO approach is apparent: for the PW6 functional there is an improvement of 1 order of magnitude in the mean absolute percentage error (MAPE) for all the rotational constants, while for the rDSD functional the relative errors are reduced by a factor of 4 for the *B*_0_ and *C*_0_ rotational constants and by an order of magnitude in the case of the *A*_0_ constant.

**Table 6:** Equilibrium and Ground State Rotational Constants (MHz) of CHBrF_2_ Obtained from the Bare Functionals (PW6 and rDSD) and after the Nano-LEGO Correction (NL)

	PW6	PW6+NL	rDSD	rDSD+NL	Exp.^a^
*A*_*e*_	10148.99	10271.35	10188.87	10251.29	–
*B*_*e*_	2883.58	2917.02	2889.04	2906.33	–
*C*_*e*_	2346.74	2374.20	2351.15	2365.32	–
*A*_0_	10089.87	10212.23	10127.11	10189.53	10199.69
*B*_0_	2873.27	2906.70	2878.24	2895.53	2903.41
*C*_0_	2336.04	2363.49	2340.03	2354.20	2360.15
MAPE	1.05	0.13	0.81	0.20	–

a From ref (74).

The next two molecules of the test-set are bromo-benzene and iodo-benzene (see Figure 5 for structure and atom labeling), whose rotational spectra have been reported by Peebles and Peebles^75^ and by Neill et al.,^76^ respectively.

The computed rotational constants of both molecules are compared in Tables 7 and 8 with the corresponding experimental data, whereas their geometrical parameters are collected in Tables S4 and S5 of the SI. Starting from the bare DFT results, the C–X (X = Br, I) bond length has been corrected employing the LRA parameters of Table 5, while the TMA has been adopted for the remaining part of the molecule, with benzene taken as the templating molecule. Vibrational corrections evaluated at the PW6 level have been used to obtain ground-state rotational constants. Once again, the Nano-LEGO correction reduces significantly the MAPE (two to four times) leading to final values (<0.22%) on par with those delivered at a much higher cost by state-of-the-art composite wave function methods.

**Table 7:** Equilibrium and Ground State Rotational Constants (MHz) of C_6_H_5_Br Obtained from the Bare Functionals (PW6 and rDSD) and after the Nano-LEGO Correction (NL)

	PW6	PW6+NL	rDSD	rDSD+NL	Exp.^a^
*A*_*e*_	5704.42	5727.26	5691.32	5738.84	–
*B*_*e*_	993.63	999.24	994.42	999.64	–
*C*_*e*_	846.22	850.80	846.51	851.34	–
*A*_0_	5661.88	5684.72	5648.78	5696.30	5667.75
*B*_0_	988.93	994.53	989.72	994.93	994.90
*C*_0_	841.79	846.36	842.08	846.91	846.26
MAPE	0.41	0.12	0.45	0.19	–

a From ref (75).

**Table 8:** Equilibrium and Ground State Rotational Constants (MHz) of C_6_H_5_I Obtained from the Bare Functionals (PW6 and rDSD) and after the Nano-LEGO Correction (NL)

	PW6	PW6+NL	rDSD	rDSD+NL	Exp.^a^
*A*_*e*_	5704.66	5727.56	5691.98	5744.98	–
*B*_*e*_	747.14	754.05	749.35	753.88	–
*C*_*e*_	660.62	666.32	662.17	666.43	–
*A*_0_	5663.20	5686.10	5650.51	5703.52	5669.13
*B*_0_	743.72	750.63	745.93	750.46	750.41
*C*_0_	657.32	663.03	658.87	663.13	662.64
MAPE	0.60	0.13	0.50	0.22	–

a From ref (76).

The two last molecules of the test set are 4-bromo-pyrazole and 4-iodo-pyrazole (see Figure 5 for structures and atom labeling), whose MW spectra have been analyzed a few years ago.^77^ In both cases, pyrazole has been used as the templating molecule, while the C–X (X = Br, I) bond length has been corrected by the LRA. The different equilibrium geometries can be found in Tables S6 and S7 of the SI, while the corresponding equilibrium and ground-state rotational constants are collected in Tables 9 and 10.

**Table 9:** Equilibrium and Ground State Rotational Constants (MHz) of 4-Bromo-pyrazole Obtained from the Bare Functionals (PW6 and rDSD) and after the Nano-LEGO Correction (NL)

	PW6	PW6+NL	rDSD	rDSD+NL	Exp.^a^
*A*_*e*_	9571.74	9567.16	9524.20	9568.22	–
*B*_*e*_	1266.44	1271.21	1266.40	1271.78	–
*C*_*e*_	1118.47	1122.11	1117.77	1122.57	–
*A*_0_	9492.57	9487.98	9445.03	9489.05	9481.06
*B*_0_	1261.56	1266.32	1261.51	1266.89	1268.30
*C*_0_	1113.31	1116.95	1112.62	1117.42	1118.43
MAPE	0.37	0.12	0.49	0.10	–

a From ref (77).

**Table 10:** Equilibrium and Ground State Rotational Constants (MHz) of 4-Iodo-pyrazole Obtained from the Bare Functionals (PW6 and rDSD) and after the Nano-LEGO Correction (NL)

	PW6	PW6+NL	rDSD	rDSD+NL	Exp.^a^
*A*_*e*_	9581.31	9576.51	9535.15	9579.67	–
*B*_*e*_	950.70	957.57	952.60	957.46	–
*C*_*e*_	864.90	870.53	866.08	870.46	–
*A*_0_	9502.31	9497.51	9456.16	9500.68	9495.62
*B*_0_	947.31	954.18	949.21	954.07	955.21
*C*_0_	861.27	866.90	862.45	866.83	867.76
MAPE	0.55	0.08	0.55	0.09	–

a From ref (77).

While the predictions of the bare functionals are already in good agreement with the rotational constants determined experimentally^77^ (MAPEs within 0.55%), the Nano-LEGO correction provides a significant improvement for all the rotational parameters. For both molecules, the Nano-LEGO corrected PW6 and rDSD equilibrium geometries yield equilibrium rotational constants that differ by 3 MHz at most, thus being essentially equivalent. Inclusion of vibrational corrections results in an excellent agreement with the experimental ground-state rotational constants, which are predicted at an accuracy within 0.12%, and the largest deviation, observed for the *B*_0_ constant of 4-bromo-pyrazole at the PW6+NL level, is 0.15%, i.e., in the range expected for state-of-the-art composite wave function metohds.^15,71^

## Conclusions

The SE100 database of accurate semiexperimental equilibrium geometries has been further extended to include systems containing Br and I atoms as well as to other molecular moieties. The resulting SE127 database allows an unbiased judgment of the performances of different model chemistries for the prediction of molecular geometries. Next, linear correlations between SE bond lengths and the corresponding values computed with hybrid (PW6) and double-hybrid (rDSD) functionals have been revised and extended to C–Br and C–I bonds. Furthermore, the previous linear regression and templating molecule approaches for the correction of computed geometrical parameters have been made more consistent in the revised Nano-LEGO tool for the prediction of accurate structures of large molecules at DFT cost. Finally, the new version of nano-LEGO has been validated against selected case studies.

The main limitations still remaining in the new tool are related, in our opinion, to the lack of an automatic selection of suitable templating molecules, to the quite high cost of the underlying quantum chemical computations for large systems (especially concerning the computation of vibrational corrections), and to the restriction to semirigid neutral molecular species containing only main group elements. Work along these lines is providing new exciting perspectives, but already the present version of Nano-LEGO can be routinely used for studying large systems of biochemical and/or technological interest.

## Supporting Information Available

Equilibrium molecular structures for organo-bromine (Table S1) and organo-iodine (Table S2) molecules belonging to the SE127 database and equilibrium geometries of bromo-difluoromethane (Table S3), bromo-benzene (Table S4), iodo-benzene (Table S5), 4-bromo-pyrazole (Table S6), and 4-iodo-pyrazole (Tables S7). (PDF)
