# Supplementary material for: Toward Spectroscopic Accuracy for the Structures of Large Molecules at DFT Cost: Refinement and Extension of the Nano-LEGO Approach
Source: J Phys Chem A. 2023 Jun 7;127(24):5183–92. doi: 10.1021/acs.jpca.3c01617 (PMC10291548; doi:10.1021/acs.jpca.3c01617)
Supplement: Supplementary file 1 — jp3c01617_si_001.pdf [file jp3c01617_si_001.pdf]

Supporting Information: Toward Spectroscopic Accuracy for the Structures of Large Molecules at DFT Cost: Refinement and Extension of the Nano-LEGO Approach

Vincenzo Barone,* Giorgia Ceselin, Federico Lazzari, and Nicola Tasinato*

Scuola Normale Superiore, Piazza dei Cavalieri 7, I-56126, Pisa, Italy

E-mail: vincenzo.barone@sns.it; nicola.tasinato@sns.it

Table S1: SE, rDSD and PW6 equilibrium geometries of organo-bromine molecules[a]

Molecule	Parameter	SE	rev-DSD	PW6
BrC≡N[b]	r(Br1=C2)	1.7888	1.7895	1.7900
	r(C2≡N3)	1.1577	1.1642	1.1594
Br_2C=O	r(O1=C2)	$1.17401^{rDSD+NLc}$	1.1775	1.1731
	r(C2−Br3)	1.9171(1)	1.9171	1.9209
	α(Br3C2O1)	123.79(1)	123.87	123.72
BrC≡CH	r(Br1−C2)	1.7897(1)	1.7921	1.7918
	r(C2≡C3)	1.2036(1)	1.2080	1.2083
	r(C3−H4)	1.0617(1)	1.0637	1.0680
BrC≡CF	r(Br1−C2)	1.7913(1)	1.7952	1.7951
	r(C2≡C3)	$1.1980^{rDSD+NLc}$	1.2002	1.2006
	r(C3−F4)	$1.2763^{rDSD+NLc}$	1.2802	1.2802
BrC≡CCl	r(Br1−C2)	1.7970(18)	1.7910	1.7909
	r(C2−C3)	1.1935(28)	1.2083	1.2086
	r(C3−Cl4)	1.6367(12)	1.6375	1.6326
BrC≡CC≡N	r(Br1−C2)	1.77982	1.7804	1.7780
	r(C2≡C3)	1.2073	1.2130	1.2128
	r(C3−C4)	1.37312	1.3728	1.3695
	r(C4≡N5)	1.15980	1.1665	1.1618
CH_3Br[d]	r(C1−H2)	1.0825	1.0853	1.0893
	r(C1−Br5)	1.9340	1.9411	1.9440
	α(H2C1H3)	111.17	111.12	111.14
CH_2Br_2	r(C1−Br2)	1.92178(14)	1.9277	1.9323
	r(C1−H4)	1.08035(54)	1.0831	1.0874
	α(Br2C1Br3)	112.862(13)	112.94	113.44
	α(H4C1H5)	112.412(84)	112.37	112.50

CH_2BrF^e	r(C1−H2)	1.08333	1.0862	1.0906
	r(C1−Br4)	1.92854	1.9361	1.9383
	r(C1−F5)	1.35757	1.3617	1.3649
	α(H2C1Br4)	107.233	107.22	107.41
	α(Br4C1F5)	110.151	110.27	110.07
$CH_2{=}CHBr^f$	r(C1=C2)	1.3256	1.3269	1.3245
	r(C1−Br3)	1.8835	1.8883	1.8922
	r(C1−H4)	1.0780	1.0808	1.0852
	r(C2−H5)	1.0794	1.0818	1.0857
	r(C2−H6)	1.0804	1.0834	1.0874
	α(C2C1Br3)	122.62	122.90	123.19
	α(C2C1H4)	124.34	124.14	124.38
	α(C1C2H5)	122.03	122.22	122.53
	α(C1C2H6)	119.28	119.29	119.25
CH_3CH_2Br	r(Br1−C2)	1.9479(1)	1.9550	1.9629
	r(C2−C3)	1.5098(1)	1.5136	1.5071
	r(C3−H4)	1.0911(1)	1.0940	1.0970
	r(C2−H5)	1.0844(1)	1.0873	1.0909
	r(C3−H7)	1.0868(1)	1.0904	1.0933
	α(C3C2Br1)	111.01(1)	111.01	111.51
	α(H4C2C3)	109.080(12)	109.39	109.14
	α(H5C2C3)	112.60(1)	112.52	112.61
	α(H7C3C2)	109.080(12)	109.39	109.14
	δ(H5C2C3Br1)	117.85(1)	117.86	117.88
	α(H7C3C2H4)	119.63(1)	119.66	119.47
$(CH_3)_2CHBr$	r(Br1−C2)	1.96334(29)	1.9702	1.9845
	r(C2−H3)	1.08761(64)	1.0892	1.0924

r(C2−C4)	1.51247(24)	1.5163	1.5106
r(C4−H6)	1.09098(98)	1.0945	1.0973
r(C4−H8)	1.0883(11)	1.0899	1.0923
r(C4−H10)	1.08867(61)	1.0917	1.0943
α(Br1C2H3)	103.584(41)	103.389	102.756
α(Br1C2C4)	108.962(16)	108.952	108.953
α(C2C4H6)	109.07(12)	109.206	109.067
α(C2C4H8)	111.321(72)	111.441	111.490
α(C2C4H10)	110.309(30)	110.558	110.856
δ(C4C2Br1H3)	118.01(24)	117.61	117.95
δ(C2C4H6Br1)	-177.80(20)	-177.92	-177.40
δ(H8C4C2H6)	-119.94(23)	-119.15	-119.20
δ(H10C4C2H6)	119.24(20)	119.71	119.95

[a] Bond lengths in Å, angles in degrees. [b] From Ref.[S1] [c] Fixed to rDSD + Nano-LEGO value. [d] From Ref.[S2] [e] From Ref.[S3] [f] From Ref.[S4]

Table S2: SE, rDSD and PW6 equilibrium geometries of organo-iodine molecules [a]

**Molecule**	**Parameter**	**SE**	**rev-DSD**	**PW6**
IC≡N[b]	r(I1=C2)	1.9921	1.9964	1.993
	r(C2≡N3)	1.1604	1.1651	1.1601
IC≡CH[c]	r(I1−C2)	1.9891	1.9927	1.9941
	r(C2≡C3)	1.2058	1.2105	1.2102
	r(C3−H4)	1.0624	1.0643	1.0687
CH_3I[c]	r(C1−H2)	1.0819	1.0851	1.0891
	r(C1−I5)	2.1336	2.1412	2.1499
	α(H2C1H3)	111.4	111.30	111.34
CH_2IF[d]	r(C1−H2)	1.0822	1.0862	1.0906
	r(C1−I4)	2.1397	2.1476	2.1554
	r(C1−F5)	1.3616	1.3642	1.3664
	α(H2C1I4)	107.00	106.96	107.17
	α(I4C1F5)	110.47	110.68	110.36
CH_2=CHI[e]	r(C1=C2)	1.3276	1.3284	1.3253
	r(C1−I3)	2.0830	2.0901	2.0990
	r(C1−H4)	1.0780	1.0808	1.0852
	r(C2−H5)	1.0794	1.0818	1.0857
	r(C2−H6)	1.0804	1.0834	1.0874
	α(C2C1Br3)	122.62	122.90	123.19
	α(C2C1H4)	124.34	124.14	124.38
	α(C1C2H5)	122.03	122.22	122.53
	α(C1C2H6)	119.28	119.29	119.25
$(CH_3)_2CHI$	r(I1−C2)	2.1670(7)	2.1747	2.1976
	r(C2−H3)	1.0869(19)	1.0891	1.0922
	r(C2−C4)	1.5162(6)	1.5179	1.5111

r(C4−H6)	1.1129(18)	1.0953	1.0983
r(C4−H8)	1.0785(16)	1.0900	1.0924
r(C4−H10)	1.0916(12)	1.0918	1.0944
α(I1C2H3)	102.73(10)	102.671	101.943
α(I1C2C4)	109.342(34)	109.367	109.219
α(C2C4H6)	106.63(19)	109.023	108.881
α(C2C4H8)	112.86(17)	111.766	111.784
α(C2C4H10)	110.598(67)	110.807	111.116
δ(I1C2C4H3)	117.919(41)	117.790	117.456
δ(C2C4H6I1)	-174.69(25)	-177.887	-177.437
δ(C2C4H8H6)	-118.91(17)	-119.022	-118.955
δ(C2C4H10H6)	122.02(26)	119.891	119.643

[a] Bond lengths in Å, angles in degrees. [b] From ref.[S1] [c] From ref.[S2] [d] From Ref.[S5] [e] From Ref.[S6]

Table S3: Equilibrium geometry of $CHBrF_2$ obtained from the bare functionals (PW6 and rDSD) and after the Nano-LEGO correction (NL)[a]

**Parameter**	**PW6**	**rDSD**	**PW6+NL**	**rDSD+NL**
r(C−Br)	1.9348	1.9337	1.9238	1.9278
r(C−H)	1.0919	1.0869	1.0855	1.0843
r(C−F)	1.3433	1.3396	1.3353	1.3355
α(HCBr)	109.16	108.73	109.16	108.73
α(FCBr)	109.59	109.66	109.59	108.66
α(HCF)	110.18	110.23	110.18	110.23

[a] Bond lengths in Å, angles in degrees.

Table S4: Equilibrium geometry of C_6H_5Br obtained from the bare functionals (PW6 and rDSD) and after the Nano-LEGO correction (NL)[a]

Parameter	PW6	rDSD	PW6+NL	rDSD+NL
r(Br1−C2)	1.9011	1.8958	1.8925	1.8911
r(C2C3)	1.3890	1.3913	1.3888	1.3890
r(C3C5)	1.3914	1.3937	1.3912	1.3913
r(C5C7)	1.3911	1.3934	1.3909	1.3910
r(C3H8)	1.0852	1.0821	1.0781	1.0783
r(C5H10)	1.0868	1.0835	1.0797	1.0797
r(C7H12)	1.0862	1.0830	1.0791	1.0792
α(Br1C2C3)	119.21	119.26	119.21	119.26
α(C2C3C5)	118.82	118.91	118.69	118.88
α(C3C5C7)	120.53	120.45	120.39	120.42
α(C2C3H8)	120.28	120.19	120.24	120.19
α(C3C5H10)	119.29	119.38	119.26	119.37
α(C5C7H12)	120.14	120.10	120.11	120.10

[a] Bond lengths in Å, angles in degrees.

Table S5: Equilibrium geometry of C_6H_5I obtained from the bare functionals (PW6 and rDSD) and after the Nano-LEGO correction (NL)[a]

Parameter	PW6	rev-DSD	PW6+NL	rev-DSD+NL
r(I1−C2)	2.1100	2.0993	2.0939	2.0928
r(C2C3)	1.3904	1.3933	1.3902	1.3910
r(C3C5)	1.3919	1.3940	1.3917	1.3917
r(C5C7)	1.3909	1.3932	1.3907	1.3909
r(C3H8)	1.0853	1.0822	1.0782	1.0785
r(C5H10)	1.0869	1.0836	1.0798	1.0798
r(C7H12)	1.0863	1.0831	1.0792	1.0793
α(I1C2C3)	119.35	119.45	119.35	119.45
α(C2C3C5)	118.98	119.12	118.85	119.09
α(C3C5C7)	120.51	120.44	120.38	120.41
α(C2C3H8)	120.53	120.42	120.49	120.42
α(C3C5H10)	119.28	119.37	119.25	119.37
α(C5C7H12)	120.14	120.11	120.11	120.11

[a] Bond lengths in Å, angles in degrees.

Table S6: Equilibrium geometry of 4-bromo-pyrazole obtained from the bare functionals (PW6 and rDSD) and after the Nano-LEGO correction (NL)[a]

**Parameter**	**PW6**	**rev-DSD**	**PW6+NL**	**rev-DSD+NL**
r(N1N2)	1.3369	1.3447	1.3429	1.3431
r(N2C3)	1.3261	1.3332	1.3278	1.3282
α(N1N2C3)	104.78	104.54	104.60	104.56
r(C3C4)	1.4075	1.4088	1.4067	1.4064
α(N2C3C4)	110.81	110.91	110.91	110.97
r(C5N1)	1.3509	1.3549	1.3510	1.3515
α(C5N1N2)	113.65	113.59	113.49	113.49
r(H6N1)	1.0058	1.0054	1.0020	1.0021
α(H6N1N2)	118.97	118.89	118.85	118.84
r(H7C3)	1.0812	1.0780	1.0751	1.0755
α(H7C3N2)	120.64	120.56	120.53	120.54
r(C4Br8)	1.8732	1.8691	1.8666	1.8652
α(Br8C4C3)	127.62	127.54	127.62	127.54
r(C5H9)	1.0798	1.0769	1.0738	1.0741
α(H9C5N1)	123.03	122.97	122.84	122.87

[a] Bond lengths in Å, angles in degrees.

Table S7: Equilibrium geometry of 4-iodo-pyrazole obtained from the bare functionals (PW6 and rDSD) and after the Nano-LEGO correction (NL)[a]

**Parameter**	**PW6**	**rev-DSD**	**PW6+NL**	**rev-DSD+NL**
r(N1N2)	1.3376	1.3453	1.3436	1.3437
r(N2C3)	1.3258	1.3329	1.3275	1.3279
α(N1N2C3)	104.73	104.48	104.55	104.50
r(C3C4)	1.4093	1.4110	1.4085	1.4086
α(N2C3C4)	111.02	111.15	111.12	111.21
r(C5N1)	1.3504	1.3543	1.3505	1.3509
α(C5N1N2)	113.58	113.52	113.42	113.42
r(H6N1)	1.0059	1.0055	1.0021	1.0022
α(H6N1N2)	118.95	118.88	118.83	118.83
r(H7C3)	1.0814	1.0782	1.0753	1.0757
α(H7C3N2)	120.43	120.35	120.32	120.33
r(C4Br8)	2.0765	2.0683	2.0635	2.0624
α(Br8C4C3)	127.86	127.82	127.86	127.82
r(C5H9)	1.0800	1.0772	1.0740	1.0744
α(H9C5N1)	122.78	122.72	122.59	122.62

[a] Bond lengths in Å, angles in degrees.

References

(S1) Le Guennec, M.; Wlodarczak, G.; Chen, W.; Bocquet, R.; Demaison, J. Rotational spectrum and equilibrium structure of cyanogen bromide. *J. Mol. Spectrosc.* **1992**, *153*, 117–132.

(S2) Demaison, J.; Margulès, L.; Boggs, J. E. The Equilibrium C–Cl, C–Br, and C–I Bond Lengths from Ab Initio Calculations, Microwave and Infrared Spectroscopies, and Empirical Correlations. *Struct. Chem.* **2003**, *14*, 159–174.

(S3) Puzzarini, C.; Cazzoli, G.; Baldacci, A.; Baldan, A.; Michauk, C.; Gauss, J. Rotational spectra of rare isotopic species of bromofluoromethane: Determination of the equilibrium structure from ab initio calculations and microwave spectroscopy. *J. Chem. Phys.* **2007**, *127*, 164302.

(S4) Zvereva-Loëte, N.; Demaison, J.; Rudolph, H. Ab initio anharmonic force field and equilibrium structure of vinyl bromide. *J. Mol. Spectrosc.* **2006**, *236*, 248–254.

(S5) Puzzarini, C.; Cazzoli, G.; Lòpez, J. C.; Alonso, J. L.; Baldacci, A.; Baldan, A.; Stopkowicz, S.; Cheng, L.; Gauss, J. Rotational spectra of rare isotopic species of fluoroiodomethane: Determination of the equilibrium structure from rotational spectroscopy and quantum-chemical calculations. *J. Chem. Phys.* **2012**, *137*, 024310.

(S6) Demaison, J. Ab initio anharmonic force field and equilibrium structure of vinyl fluoride and vinyl iodide. *J. Mol. Spectrosc.* **2006**, *239*, 201–207.
